# Social media as societal microcosm: A decade of LGBT Twitter conversations in Singapore

**DOI:** 10.1371/journal.pone.0332700

**Published:** 2025-10-09

**Authors:** Reuben Ng, Ting Yu Joanne Chow, Wenshu Yang

**Affiliations:** 1 Lee Kuan Yew School of Public Policy, National University of Singapore, Singapore, Singapore; 2 Lloyd’s Register Institute for the Public Understanding of Risk, National University of Singapore, Singapore, Singapore; Northeastern University, UNITED STATES OF AMERICA

## Abstract

**Background:**

Singapore occupies a curious societal grey-area: a digitally savvy country with a colonial-remnant law against homosexuality (penal code 377A), widely acknowledged as non-proactively enforced, existing to placate a conservative society; hotly contested for years and finally repealed in Parliament in 2022. Within a national context of state-upheld heteronormativity, yet with homosexuality not entirely condemned, Singapore occupies a liminal space where subtle resistance is carefully negotiated, especially in online spaces.

**Objectives:**

This study investigated LGBT-adjacent discussions across social media over a decade (2011–2021) for salient topics, sentiment distribution, emotional intensity frames and nuanced topics.

**Methods:**

Historical Twitter data containing LGBT keywords (N = 15,659) were collected and analyzed using bi-term topic modelling, sentiment score modelling, and emotional intensity modelling. Qualitative thematic analysis was conducted on highest-scoring emotion tiers.

**Results:**

Sentiment was distributed over a range: Very Positive (6%), Positive (33%), Neutral (11%), Negative (42%), Very Negative (7%). Predominant emotions were Joy (39%), Anger (32%), Sadness (11%), Fear (11%). Of themes from highest-scoring emotional-intensity tweets, Anger included: ‘gay’ used derogatorily; heated debates over ideological—often religious—differences; dissent within the community, condemning exclusionary views. Fear and Sadness included distress over violence (mass shootings, harassment, bullying); lack of acceptance (criminalization, protests over local pride event ‘Pinkdot’; lack of familial support). Joy stemmed from the celebration of pride month.

**Conclusions:**

Our findings highlight nuanced emotional intensities, profiles undertones of LGBT dissent and support, fractured along a schism of differing views and contrasting opinions—a societal microcosm of a divisive topic. Practically, this presents a decade-long barometer of dominant trigger points that may help facilitate conversations on the affective concerns of the local population.

## Introduction

The impetus for this study was sparked by Singapore’s curious occupation of a societal grey-area: a digitally savvy country with a law against homosexuality, yet having the law widely acknowledged as a tenuously-enforced one, existing to placate conservative sections of society. The law in question, Penal Code Section 377A (colloquially, 377A) was hotly contested between 2007–2022, before finally being repealed by Singapore’s Parliament on 29 November 2022 [1]. The inciting incident bringing this law to the fore occurred in 2007, where Penal Code 377 (a law prohibiting *heterosexual* partners from consensual sexual acts of fellatio and anal sex, a remnant from colonial-era British rule was repealed), but 377A (a law prohibiting *homosexual* male partners from consensual sexual acts of fellatio and anal sex) deliberately retained [2]. Despite this ruling, the Singaporean Prime Minister conceded that 377A was not to be proactively enforced [3]. Following this, the intervening years saw a struggle for repeal [4] and the establishing of *Pink Dot* as a local pride event in 2009: the event gaining traction over the decade, though met with polarized resistance from protestors of multiple religious communities [5,6]. Within a national context where heteronormativity was upheld by the state, yet homosexuality is not fully condemned, Singapore occupies a liminal space where subtle resistance is carefully negotiated by LGBT groups [7]. The liminality of existence stems from living in a predominantly heteronormative public sphere: the local Pink Dot pride event being the one exception held at the only public space in the country which allows for a mass assembly [8]; Singaporean scholars have cited further that non-normative sexualities are repressed by the state using legal and social levers [9], with tolerance applied to some spheres at best [10]. These sociopolitical dynamics thus provide a unique lens into which queer visibility and agency is navigated and negotiated within constrained civic spaces, when considering that pride events may engender societal backlash, yet fluctuations in broader political developments and social norms have evidently softened the extent of explicit discrimination faced by the individual. It is therefore worth mapping the subtle dialectic between institutional containment and grassroots expressions of belonging; an approach that captures the discursive ambivalences of queerness and its associated textual contexts of everyday speech.

With this context established, this study analyzed historical Twitter data in Singapore, specifically about LGBT (the lesbian, gay, bisexual, and transgender)-related conversations over a decade (2011–2021). The study’s span is of particular interest, given that it is a decade bookended by 377 A’s shadow: from its point of legal contention to it ultimately being repealed. Specifically, this study asked the following two exploratory questions. The first core question asks (i) What are the most common topics discussed in relation to LGBT-adjacent conversations on Twitter? The second core question asks:(ii) What are the emotional themes associated with LGBT conversations? Are they more negative or positive in valence? What types of emotions are associated with these conversations—anger, fear, sadness, or joy?

The utility of using social media to understand LGBT issues on a societal level is demonstrated by several studies that have made use of such explorative methodologies. Several studies make use of large-scale historical Twitter data to investigate broader narratives about LGBT issues within a community, significantly cataloguing societal opinions and gauging dominant perceptions for and against their legal rights. For instance, following India Supreme Court’s decriminalization of homosexuality—their own repeal of law 377—a study [11] collected half a million Indian tweets to gauge societal support or skepticism toward this decision; finding that tweets were split between anti-homosexuality (opposing the verdict on the grounds of ‘traditional’ and ‘cultural’ family values) and pro-homosexuality (supporting human rights and equality). We springboard from this study by postulating that large-scale national-level corpuses via the lens of social media provides insights into societal microcosm. These insights are useful for local politicians in understanding active issues with strongly differentiated views, particularly because emotions are central in how a population engages in, and reacts to, contentious politics [12,13]; a politician’s ability to capitalize on emotional capital by being in tune with dominant emotion norms is postulated to be a form of cultural capital [14]. As there has yet to be such a big-data approach applied to the Singaporean context, this study is positioned to expand the scope of investigation given similar contexts of repeal delineated above to provide novel insights specific to the local situation.

In that vein, many studies into social media yield unique and socially-incisive findings. An analysis of Twitter narratives on homosexuality in Nigeria [15] ironically blamed the government for being simultaneously too anti-homosexuality (by not protecting marginalized communities enough) and pro-homosexuality (by not being tough enough on legislation against the queer community). Such an approach can also yield a general timeline and narrative of social memories: for instance, of celebrated LGBT athletes, tracking their social impact and legacy across historical mentions on Twitter [16]. An explorative study zoomed in on content mentioning LGBT healthcare on Twitter [17], citing how tweets hold surprising potential for honest opinions about contested issues across niche topics, given the platform’s unfiltered nature [18]. The utility of social media corpuses in elucidating nuanced societal opinions is significant, as it holds space as virtual public sphere for controversial illiberal contexts [19]. Despite homosexuality being explicitly illegal in multiple other countries, LGBT communities have continued to thrive on social media, using the platform to engage and communicate within the community [20], or in representing the self [21]; emerging as a form of digital sexual citizenship where LGBT youths find friendships and kinship among peers [22], to learn from one another [23] and spread messages on equal rights [24]. The novelty of the study is therefore positioned against a context of previous social media work on LGBT discourses revealing salient representations of the self and digital kinship amidst contexts of social persecution.

Compared to the general population, members of the LGBT community are reportedly more susceptible to facing social inequality [25], stigma and discrimination [26], online hate speech [27], often having ramifications on mental and physical welfare: translating to increased risk factors for mental health issues [28] and healthcare hurdles [29], presenting crucial impetus for policymakers to address; yet attention and research into this demographic has only gained more traction in recent years [30] and across mainstream discourses [31]. These negative effects are particularly heightened in the context of a culturally conservative Southeast Asia, with political and social stressors that affect LGBT individuals more than cisgender or heterosexual people: a systematic review of quantitative studies about LGBT Southeast Asians indicate a higher prevalence of mental health difficulties [29,32], and suicidal ideation in non-heterosexual Singaporean men [33]. Ethnographic interviews with gay Singaporean men revealing hesitance to ‘come out’ as gay due to fear of prosecution and discrimination [34]; and ambivalent feelings associated with marginality [35]. It is therefore crucial to examine the online space in which LGBT individuals often inhabit.

## Materials and methods

### Dataset

**Fig 1:**
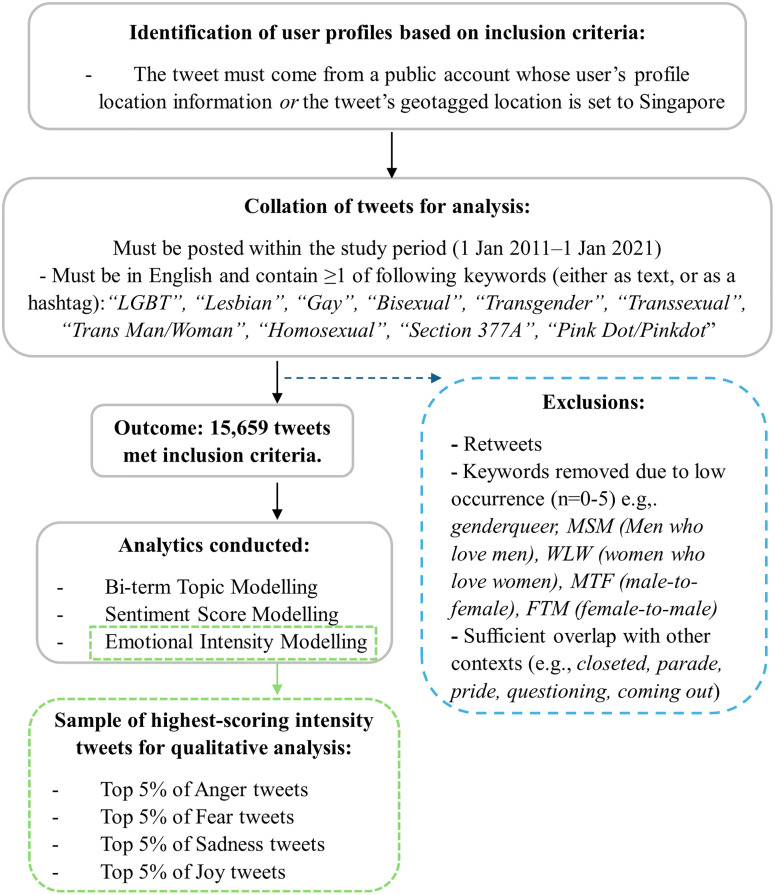
Collation of the Singapore LGBT Twitter Corpus and Analytic Strategy.

Tweets related to LGBT topics from public twitter accounts geotagged to Singapore, posted from January 1, 2011, to January 1, 2021—a decade of publicly-available data—were collected using Twitter’s application programming interface standard search, using LGBT-adjacent terms as search keywords; all publicly accessible tweets containing any of our keywords were collected for this study. The following keywords were selected: *“LGBT”, “Lesbian”, “Gay”, “Bisexual”, “Transgender”, “Transsexual”, “Trans Man/Woman”, “Homosexual”*, along with Singapore-specific keywords *“Section 377A”* (i.e., a hotly-contested, codified law prohibiting intercourse between consenting homosexual adults), “*Pink Dot/Pinkdot*” (i.e., the name of Singapore’s local pride event, held during Pride Month). We visually represent this data collation process in Fig 1.

This corpus of tweets (N = 15,659) was then used to elucidate (i) the most common topics and (ii) the nuanced emotional themes associated with LGBT conversations on social media. All usernames or identifying information was redacted prior to analysis and reflected as such in the sample tweets provided in the result section.

### Analytic strategy

(i) Topic analysis using biterm topic modelling

For topic analysis, we used Biterm Topic Modelling—a topic modelling method ideal for corpuses comprising short texts such as tweets, performed using the R package BTM—to summarize the latent topics that commonly surface in LGBT-adjacent content in Singapore, bringing to the fore the most prominent content associated with the community. The model that produced the most optimal results, evinced by the lowest perplexity score upon several iterations of collapsed Gibbs sampling, provided 25 clusters of terms. Subsequently, these topics were tagged manually by two independent researchers, and categorized into 6 meta-themes, and reported in the results section. The BTM model was selected for the analytic approach as it was designed for, and more accurate for short text classification. For instance, compared to other models like BERTopic in handling sparse semantics, BTM has proven to offer better interpretability and stability for small datasets [36] similar to the context of this study (i.e., short tweets), as it explicitly models word co-occurrence patterns on a whole-of-corpus level, rather than on the document-level.

(ii) sentiment analysis and emotional intensity modelling

To address this analytical angle, we used sentiment and emotional intensity modelling algorithm CrystalFeel [37]—a sentiment analytic method that analyses the valence (positivity/negativity) of short-form texts, and emotional intensity scores in natural language, specifically developed for social media analysis. The algorithm, trained using multiple datasets that consider linguistic markers like vocabulary, grammatical phrasing, punctuation, and emojis that people employ on social media, produces predictive scores on *what emotions are expressed in the tweet* (i.e., anger, fear, joy, sadness) *and how intense they are* (on a quantitative scale of 0–1). More specifically, each emotion is represented by a continuous variable with its value ranging from 0 to 1, where 0 refers to a barely-noticeable amount of the emotion and 1 expresses an extremely high intensity of the emotion. For instance, the emotion “fear” is psychologically understood by an unpleasant emotion arising from a perceived threat, often leading to confrontation with, avoidance of the threat. On the scoring system, a low-intensity fear-associated feeling would relate to “apprehension” or “worry” (thus scored lower on Fear intensity), while a high-intensity feeling would relate to “dread”, “horror”, or “terror” (thus scored higher on Fear intensity). This allowed for a measurement of objective intensity for four different emotional qualities.

The model was selected for its demonstrated high predictive accuracy tested against human-annotated data in predicting emotional intensity. Specifically, the predicted accuracy of the algorithm was validated based on a Pearson correlation coefficient R-value, evaluated against out-of-training test samples of human annotations, with the intensity predictors for anger, fear, sadness, joy and valence respectively being 0.814, 0.775, 0.766, 0.791 and 0.860; suggesting that the external validation statistical analysis was sufficiently robust.

Furthermore, the algorithm embeds efforts to reduce the risk of incorrect coding by considering sarcasm detection to enhance the performance of sentiment classification [38]. This was achieved using an affect-cognition-sociolinguistics sarcasm features model and a trained SVM-based classifier for detecting sarcastic expressions from general tweets; thereafter a two-level cascade classification system implemented by considering those scores alongside external lexicons, n-grams, word embedding vectors, and part-of-speech features. This optimization for low error rates in subtasks ensured tone-aware quantification performance, especially considering the context of social media linguistic style and its more casual contexts of use. This algorithm was also selected over other sentiment analysis models given its robustness at the time of writing, particularly in deriving not only emotions (anger, fear, joy, sadness) but also their intensity on a continuous scale; an aspect mostly absent in short-text models that more commonly only allowed for categorical scoring (positive vs neutral vs negative). As the topic of study deals with multi-faceted human emotions, it was crucial to adopt an algorithm to sufficiently differentiate between higher-intensity and lower-intensity emotional expressions. For example, anger expressed in a tweet could run from low-intensity annoyance (e.g., from being irritated by an off-hand comment about homosexuality) to high-level fury (e.g., in response to hate crimes and violence experienced); thus, intensity ratings provide further context for how the studied LGBT issues have been spoken about in social media spaces.

We report the findings on LGBT tweet valence and emotions, as well as further sampled the top 5% of tweets that scored the highest on emotional intensity (i.e., the top 5% of angriest tweets, top 5% of fearful tweets, top 5% of joyful tweets, and top 5% of sad tweets). This sampled data sub-set was then reviewed and categorized qualitatively by two independent researchers, assessing the nuanced themes surrounding anger, fear, joy, and sadness related to LGBT conversations. This qualitative dimension of analysis served dual purposes: first, to provide substantive context for the emotional scores, and second, to validate the scores using manual verification. During manual tagging, it was found its accuracy cohered with algorithmic scoring, validating its strong prediction performance (0.816 sentiment intensity in Pearson correlations on gold test data). For full data transparency, the supplementary material section for this study contains a sample of these tweets, along with their assigned emotion intensity ratings.

## Results

### Overview and summary

Our analytic foray into LGBT-related tweets in Singapore over a decade (2011–2021, n = 15,659)—against a social backdrop bookended by a controversial yet non-enforced law penalizing non-heterosexual acts of intimacy—revealed interesting insights into the topics, valence, and nuanced emotional themes that Singaporeans discuss in relation to LGBT-adjacent conversations on social media.

To summarize, the following insights were gleaned: (i) Through topic analysis using Biterm topic modelling methodology, we found that the most statistically likely themes that cluster together within our corpus were: conversational content (31.3%), support within the LGBT community (25.9%), LGBT Pride (16.1%), TV, Film and Pop Culture (12.3%), Lifestyle, Leisure, and Fashion (8.6%), Fandom (5.8%). Our findings from (i) suggest that, statistically speaking, based on unique latent topics in the corpus, Singaporean LGBT users tend to use Twitter as a space to discuss lifestyle content and find support within the community. This coheres with past studies on other LGBT communities that demonstrate social media as a key source for garnering social support [39]; the presence of quotidian and daily topics reflective of the space allowing for open self-disclosure [40]. The ‘everyday’ topics signals a discursive normalization of queer identities that transcends overt activism, aligning with findings that such digital spaces facilitate open expression and identity affirmation, while the pride-related topics signals the dual refuge of online spaces to build resilient networks of belonging.

**Fig 2:**
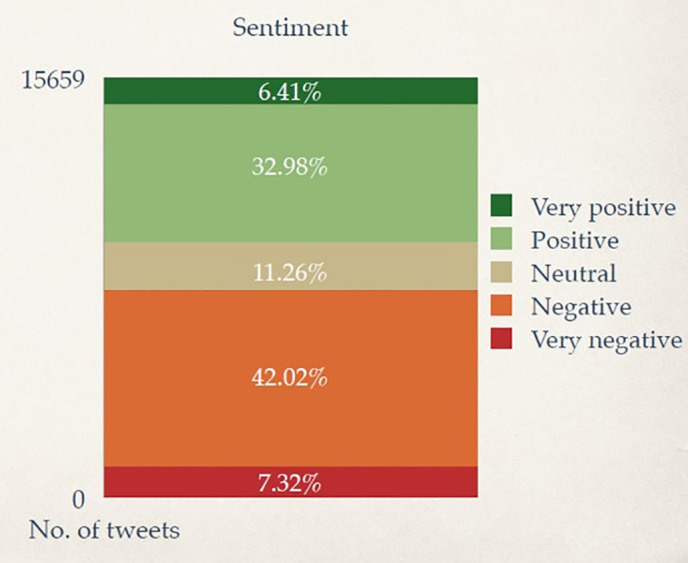
Sentiment valence of LGBT-related Tweets from 2011 to 2021.

**Fig 3:**
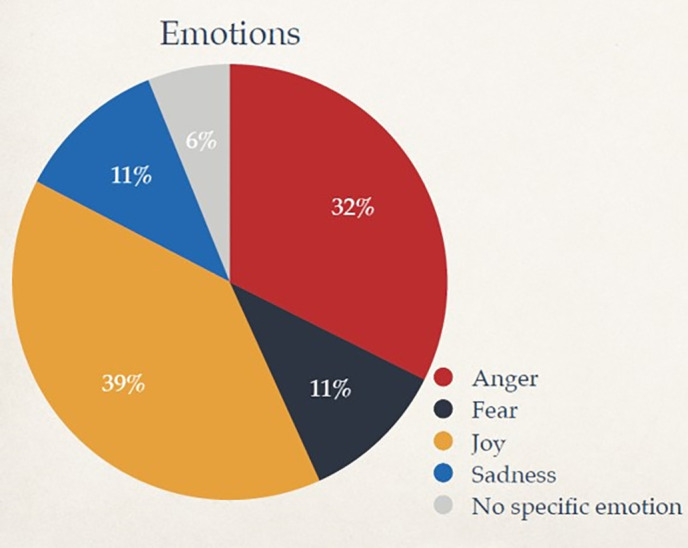
Overall emotional profile of LGBT-related Tweets (2011 to 2021).

**Fig 4:**
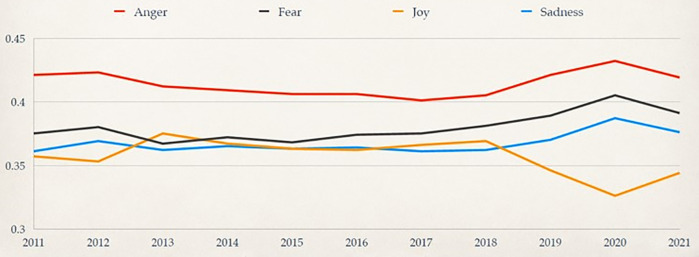
Comparison of emotional ratings of LGBT-related Tweets from 2011 to 2021.

Next, (ii) Through nuanced emotional thematic analysis using CrystalFeel, a sentiment and emotional intensity model, we found (a) the following quantitative metrics. Out of all the LGBT-related tweets in our corpus posted between 2011–2021, the sentiment valence was as follows (Fig 2): Very Positive (6.41%), Positive (32.98%), Neutral (11.26%), Negative (42.02%), Very Negative (7.32%). The predominant emotional profile of the corpus was as follows (Fig 3): Joy (39%), Anger (32%), Sadness (11%), Fear (11%), No specific emotion (6%).. The annual distribution of predominant emotions (Fig 4) found that Joy exhibited the biggest on the biggest year-on-year decline, reaching its lowest percentage presence in 2020, while Anger, Fear and Sadness peaked in that same year. Together, findings from (ii)(a) suggest it is equal parts heartening and disconcerting that positivity/joy and negativity/anger are exhibited in the corpus, symbolic of the topic being a divisive inflection point. On a macro-view, the presence of conflicting, yet co-existing emotions and discourses found in the LGBT corpus dataset reflects a diversity of opinion made by different locals at different points in their life and represents differing viewpoints that circulate the online social space regarding this social group. On a year-on-year view, the declining presence of Joy and increasing presence of negative emotions warrants concern.

We further delved into the top 5% of tweets with the highest emotional intensity scores (anger, fear, sadness, joy) to find (b) the following qualitative emotional nuances. Of the corpus content with the highest anger-intensity scores, ‘gay’ was commonly used as a derogatory term, alongside heated debates over ideological—and often religious—differences, both for and against the right for LGBT identities to co-exist within the framework of society. A smaller handful of tweets also reveal dissent even within the LGBT community, particularly directed toward individuals who hold transgender-exclusionary views. Anger was therefore nuanced along a schism of many viewpoints and contrasting opinions. Of the corpus content with the highest fear- and sadness- intensity scores, we found that the most common contributors to such expressions of distress were over real-life violence (e.g., the mass shooting at a gay nightclub in Orlando in 2016; various news reports of LGBT individuals being harassed or beaten up by assailants), the lack of social acceptance (e.g., the continued criminalization of homosexuality in multiple countries across Asia; protests against Pinkdot; the lack of familial support), and being bullied for one’s sexual orientation. Of the corpus content with the highest joy-intensity scores, the main source of happiness and warmth stemmed from the presence of Pinkdot and community support during pride month. Our findings from (ii)(b) surfaced complex issues, with each sub-theme carrying the potential to be further problematized and examined in closer detail.

(i) **Topic analysis**

In Table 1, we detail the results of bi-term topic modelling. Unsurprisingly, owing to the conversational nature of Twitter, the most common topic was conversational content, containing casual internet lingo like *lol, haha, omg*. This topic also contains words that connote to romance or interpersonal ties, such as *love, couple, cute, song, friend.*

**Table 1:** Topic analysis of LGBT-related tweets over 10 years (2011-2021).

Topic	Share	Terms
Conversational Content	31.3%	*gay, fuck, hahah, asia, im, lol, la, lesbian, do, haha, guy, ah, damn, wtf, boy, couple, straightfriend, straight, cute, update, instagram, post, omg, friend, love, song*
Support in the Community	25.9%	*singapore, lgbt, lgbtq, support, community, right, homophobia, issue, marry, ideology, normal, queer, love, hope, space, friend, happy, birthday, cake, talk, partnership*
LGBT Pride	16.1%	*pink, dot, pinkdot, pinkdotsg, hong, lim, park, singapore, love, support, freedom, freedomtolove, pride, happy, lgbt, marriage, transgender, trans, support, gaypride, gaylove, equality, honglimpark, pinkdotsg2019, loveislove, pinkdot11, loveforppl, loveconquersall, discrimination, pinkdot2019, loveforpeople, loveisnotimmoral*
TV, Film, Pop Culture	12.3%	*queer, eye, drag, gay, trans, lesbian, movie, video, play, love, lgbt, actor, woman, guy, role, lesbian, bisexual, people, character, trans, set, lesbian, race*
Lifestyle, Leisure, Fashion	8.6%	*club, tabooclubsg, night, taboosg, welovetaboo, music, party, bar, taboo, deejay, dance, Saturday, mensfashion, fashionblogger, menswear, singapore, fashiondesigner, luxurylifestyle, trans, makeupartist, model, fitness, lesbian, beauty, beautiful, wanderlust, adventure, explore, gaytraveler, travelgram, travelphotography, vacation, globetrotter*
Fandom	5.8%	*nct127, nct, hyung, jhope, blackpink, wonshik, trans, heart, girl, jimin, ikon, lgbt, read, base, argue, call, yg, transgender, book, support, ha, fan, kimj1won*

The second-most common topic involves warm community-based words such as *support, community, love, hope, friend, space*; and marriage-adjacent terms like *marry, partnership, right* and words about discrimination: *homophobia, issue, ideology.* Within these contexts, it is likely that conversations are being had about seeking support and a safe space within the LGBT community.

The third-most common topic revolves around pride event Pink Dot, surfacing wholesome collocations like *freedomtolove, gaypride, gaylove, loveislove, loveforppl, loveconquersall, loveforpeople, loveisnotimmoral*; annual hashtags for the event: *pinkdotsg2019, pinkdot11, pinkdot2019, pinkdot, pinkdotsg*; geographical words like *hong, lim, park, honglimpark*, detailing the location of annual Pinkdot events.

The fourth-most common topic likely involves television and pop culture, with *queer eye* and *drag* in this list of terms, probably due to the popularity of the television programme of that name. Other showbiz related terms include *movie, role, actor, video, character, set*, likely stemming from discussions about films and shows that portray LGBT characters and stories.

The fifth-most common topic contains varied lifestyle and leisure activities, such as the inclusion of popular local gay nightclub: *taboosg*, *welovetaboo, club, tabooclubsg, night, music, party, bar, taboo, deejay, dance, Saturday*; fashion and makeup terms are also present: *mensfashion, fashionblogger, menswear, fashiondesigner, makeupartist, model, fitness, beauty, beautiful;* alongside travel terms *wanderlust, adventure, explore, gaytraveler, travelgram, travelphotography, vacation, globetrotter luxurylifestyle.* Lastly, a small percentage of terms revolve popular Korean-pop artistes: *nct127, nct, hyung, jhope, blackpink, wonshik, jimin, ikon, yg*, likely due to typical fandom behaviour involving fan speculation on the sexuality of celebrities, or pairing two compatible artistes together.

(ii) **Nuanced emotional themes**

### Sentiment Valence and predominant emotions

In Fig 2, we present the results of our sentiment scoring model, highlighting that valence share was generally evenly distributed between positive and negative. Specifically, sentiment valence was overall: Very Positive (6%), Positive (33%), Neutral (11%), Negative (42%), Very Negative (7%). In Fig 3, we present the results of our emotional scoring model, highlighting that the predominant emotional profile was: Joy (39%), Anger (32%), Sadness (11%), Fear (11%), No specific emotion (6%). In Fig 4, we provide emotional intensity results of our emotional modelling over the last decade, finding that the most viscerally expressed emotion in the LGBT Singapore Twitter corpus was Anger (i.e., had the strongest overall scores), followed by Fear, Sadness, and Joy. This figure also charts the shifts in predominant emotions based on annual distributions.

### Thematic analysis of anger, fear, sadness, and joy

**Fig 5:**
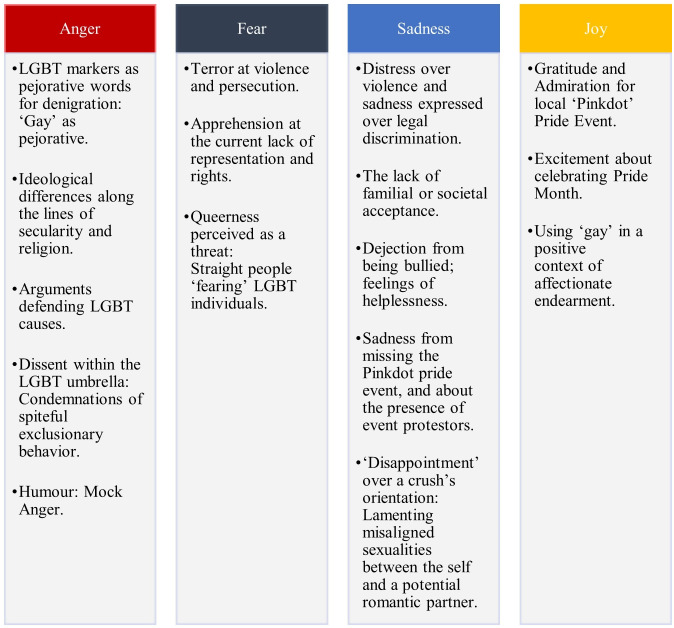
Emotional profiles and topics of LGBT-related Tweets from 2011 to 2021.

In Fig 5, we provide an overview of the nuanced themes associated with the strongest emotional intensity. A full summary of tweet samples, with emotion intensity ratings, and the year the tweet was made may also be found as supplementary material for this study.

### Topics of anger-related tweets

Commonly, markers of sexual identity were used pejoratively by Singaporean social media users and used as a derogative descriptor. Most commonly, words like ‘gay’, ‘transgender’, ‘queer’ and ‘fag’ were used in the contexts of denigration and the expression of disgust, toward another person, activity, or object. The use of identity markers as hurtful insults may be problematized for its potential hurtfulness on individuals who do identify as LGBTQ. Controversially, the top anger-intensive content in the corpus was also rife with fights over ideological differences, expressed predominantly by a perceived tension between secularity and religion.

Conversely, common themes also include LGBT supporters rallying against homophobia, with posters displaying a strong sense of defensiveness for their peers and exasperation directed toward various levels of society. Often, this presented as anger directed toward homophobic behavior, ranging from bullying to hateful comments in online spaces. Criticism of implicit homophobia encoded in stereotypical depictions of gay and transgender characters in local television programs were present, critiquing Singapore’s lack of LGBT inclusivity in localized media. Outrage was also expressed over others who openly disparage local pride event Pinkdot, real-world violence enacted upon LGBT spaces. High-anger-intensity datapoints also indicated dissent from within the LGBT community, with individuals calling out problematic behaviors (e.g., condemning those holding spiteful transgender-exclusionary viewpoints, those who promote negativity online under the guise of inclusivity).

On a lighter note, a handful of posts sometimes leveraged LGBT markers in humorous contexts. For instance, declarations like ‘this is homophobic’ were commonly attested in situations of trivial inconveniences, used for dramatic effect to communicate humor on the internet. In other contexts, ‘gay’ was also used as an intensifier for inconsequential situations made in jest.

### Topics of fear-related tweets

Upsettingly, the top fear-intensive content in the corpus contained fears of violence and persecution: being followed, physically threatened, doxed (i.e., victim of personal identifying information maliciously leaked online), killed, or arrested. These fears are rooted in real-world occurrences of LGBT individuals being persecuted for their sexual identities, either by codified law, homophobic individuals, or targeted mass shootings that occurred beyond the local context. Also present were fears over potentially losing one’s friends upon revealing their sexual identity, and concern over the sobering reality of higher suicide rates and mental health issues in non-heterosexual communities.

Queerness is also somewhat framed as a threat, often in the form of assumptions made by non-LGBT tweeters. For instance, a common theme involved finding out an individual in one’s social circle was non-heterosexual, an assumptive leap made toward automatically being considered a ‘target’ or ‘crush’ and conveying of being ‘scared’ of the LGBT individual through a tweeted anecdote. Other assumptions included fans of certain popular boy bands being labelled non-heterosexual stalkers. It is worth considering that the continuum of fears expressed by non-heterosexual tweeters, compared to heterosexual tweeters, is dichotomized: on one end of the spectrum is crippling fear over violence, persecution, and the dearth of social support; while the other caricaturizes non-heterosexual identities as inherently predatory.

### Topics of sadness-related tweets

Various shades of disgust, dread, and resignation over real-world issues that LGBT individuals face over the globe were the most common sources of sadness. The biggest sources of distress were past targeted shooting at a gay bar in Orlando; homosexual men in Singapore facing violence and discrimination for their sexuality; and legal hurdles being transgender, which tends to be highly stigmatized lived experiences fraught with legislative difficulties. Stories shared most commonly involved the lack of familial support, parental abuse, verbal disparagement and bullying, contextualized against Singaporean societal norms. Often, these shared experiences were paired with emotions like helplessness, hurt, feeling abandoned, or suicidal. Many tweets revolved around feeling disheartened about protests against local pride event Pinkdot, where negative comments about pride and active campaigns against it (i.e., a *Wear White* movement) were held.

On a comparatively light-hearted note, a handful of sadness-intense datapoints in the corpus contained individuals lamenting misaligned sexualities between the self and a potential romantic partner. Some expressed disappointment over various characters on television shows having a different sexual orientation than they originally expected.

### Topics of joy-related tweets

Overwhelmingly, the top joy-intensive content in the corpus was Pride-adjacent. Gratitude for the local ‘Pinkdot’ event was expressed in celebratory content conveying happiness, admiration, warmth, and community over several years; hearteningly, the main and largest source of joy in the corpus was facilitated by this local movement uplifting the queer community. Along a similar thread, excitement and happiness was expressed particularly during pride month, with joyous expressions about the support within and toward the community, and celebratory declarations of love during pride month.

Perhaps paradoxically and in direct contrast to ‘gay’ being used pejoratively in the highest anger-intense tweets in the corpus, a noticeable volume of tweets in the corpus ranking high on the joy scale used the word to denote genuine positivity. The word is often also used in positive contexts of affectionate endearment to describe a close friend, denote happiness, or in a complimentary context.

## Discussion

Our study was launched with the intention of understanding emotionally-charged opinions about a societally controversial topic. With the re-invigoration of 377A into public interest over the recent decade, this era is bookended by a deliberate denial of repeal in 2007 and only repealed in 2022. The emotionally-charged opinions and polarized valence of the online populace (with sentiment results being equal parts positive and negative in valence, and distribution of emotions being simultaneously joyous yet counterbalanced by narratives of anger) is reflective of a societal microcosm nuanced along a schism of various views and contrasting opinions. The divisiveness of this topic through the lens of LGBT-adjacent mentions on social media provides a baseline for further problematization. For instance, issues that have surfaced include the (paradoxical) use of ‘gay’ as a term of endearment in some local circles, but is also used as a term of denigration. It is also apparent that the fears of the LGBT community are often centered around painful incidents of violence internationally, bullying, legal discrimination, and the fear of rejection from their own family and friends. This level of divisiveness further coheres with past studies that denote the dual-edged nature of online communities, as they serve to be candid spaces for LGBT self-expression, yet also contain stress-prejudice events and perceived stigma given the wide-reaching nature of online platforms [41]. Furthermore, the increasing prevalence predominantly negative emotions like anger, sadness and fear year-on-year coheres with recent findings of increments in hate speech against LGBT individuals found online [42]. It is however, still heartening that a source of significant joy stems from the presence of Pinkdot in Singapore—the establishing of a local pride event that has gradually gained traction and injected much-needed positivity into the online sphere.Of practical significance, we provide insights into a societal microcosm, which hinges on understanding what people are feeling. These nuanced sources of emotion may be relevant to policymakers or local leaders who wish to understand the issue on a societal level, and utilize key insights into emotionally-sensitive trigger points. In our introduction literature we highlight that the savvy politician wields significant cultural capital by being in tune with the dominant emotion norms of local populations. This is particularly significant for contentious topics, where emotions take center-stage. The utility of such an approach helps facilitate conversations about the issue: being sensitive to, and addressing the emotions surrounding topics of contention allows the leader to address the affective concerns of the community they serve. In the context of citizen town halls and public communications, knowing if a population are angry, fearful, or upset—rather than hopeful—allows the public speaker to manage expectations and speak to the concerns of the people.

Of conceptual significance, this study provides an expansive analysis of LGBT narratives in Singapore on a wider scope than most existing local studies, which are often smaller in scope by using survey or focus group data. Existing investigative studies are similarly limited in scope, offering close readings of specific pieces of media coverage (such as of promotional material of anti- or pro- LGBT events), delving into the intricacies of media constructionism. By scoping our study to include *whole-of-nation* LGBT-adjacent social media mentions, we provide a different and non-restrictive lens of this issue on a larger, national scale.

**Fig 6:**
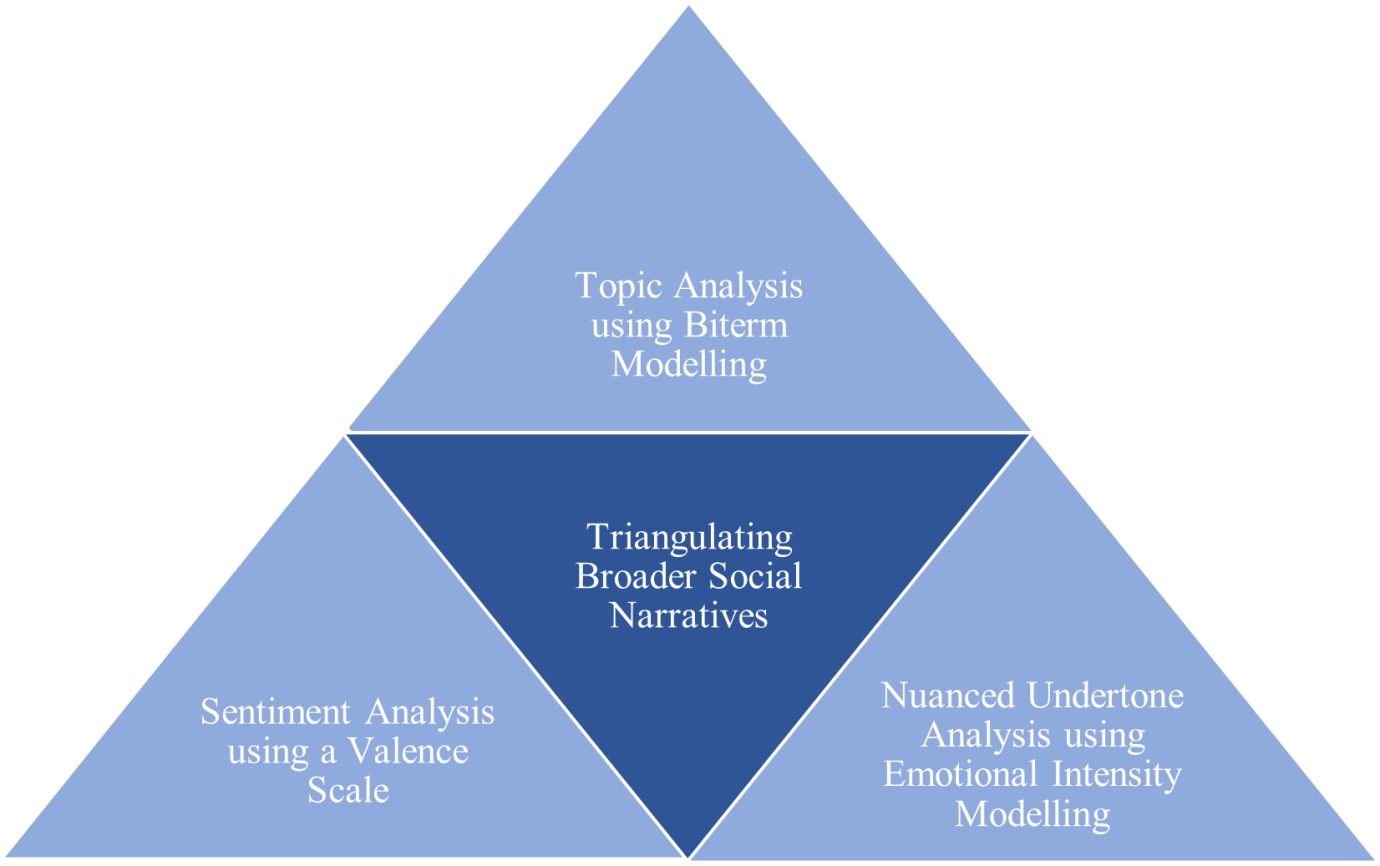
A replicable triangulated framework on the analysis of contentious social issues.

Of methodological significance, our study provides a replicable triangulated framework on the understanding and deconstruction of complex social issues through the lens of social media data. We refer to Fig 6 and the involved methodologies for our study: combined, these metrics provide the researcher with a full picture of existing social narratives within a designated time-frame. We circumvent any potential blind spots on how an issue is publicly perceived by using a three-pronged holistic approach: topic analysis provides overarching coverage of popular themes, while sentiment and emotional intensity analysis supplements these broad strokes with nuance. We posit that this provides a novel spin on the study of social issues by using emotional intensity scoring as a filter. Instead of taking a random sample of tweets to code for thematic content, we zoom in on the highest-intensity content scored by our emotional intensity model. This twist on methodology attempts to innovatively circumvent the pitfalls of existing corpus data studies that rely on sentiment scoring. This method, while useful, tends toward simplistic conclusions, as it does not reveal the full scope of a controversial issue—often, the valence score averages out to a neutral one in a corpus evenly split among supporters and detractors. Thus, by opting to zoom into the emotional undertones and understanding its nuances, our methodology captures the complex expressiveness of people’s opinions. By taking a triangulated approach, we underscore the critical significance of underlying social perceptions.

Also, of methodological and conceptual significance is the replicability of this triangulated framework for complex social media corpus data. Its immediate applicability for methodological extension is into other Commonwealth nations that share similar pasts rooted in colonial-era Britain, particularly those still host to a legacy of laws that criminalize LGBT communities. It is worth examining these nations that have also inherited anti-homosexuality laws; our framework provides an exportable template to analyze the underlying emotional undertones and intensity of social media data about LGBT-adjacent conversations.

### Limitations

This study is not without its limitations: by nature of social media, user demographics tend to skew slightly younger; thus, the results that we obtained may have unwittingly had age as an influencing factor in reflecting the sentiments of a younger societal demographic. Further, data was only obtainable for user accounts that were set to public, and specifically of user accounts whose profile location information, or tweet’s geotagged location marker, has been set to Singapore. Thus, any data from private accounts, or accounts in which geographic information is unknown, is by default omitted from the corpus. Lastly, by conducting qualitative analysis on only the top 5% of highest-scoring emotional tweets, this meant that tweets that contained less emotive language were not delved into. The views and attitudes encoded in such moderate tweets are thus not represented in our final results.

## Conclusions

This study has provided a decade’s overview of societal attitudes and emotions through the mitigating lens of Twitter social media, by studying underlying emotional undertones and intensity of LGBT-adjacent conversations. The study topic is pertinent in a country experiencing a tumultuous era involving an explicitly anti-homosexual law and its bookended journey from re-invigoration of public interest, to deliberate denial of repeal efforts, and eventual repeal. We present findings suggesting a schism within this online social microcosm: polarized valence exhibited in the online populace, and wide array of nuanced emotional concerns and contrasting opinions. Practically, this provides insight into dominant trigger points that may help facilitate conversations on the affective concerns of the local population. Conceptually, we expand existing local LGBT literature by providing a substantial data scope of over a decade. Methodologically, we provide a replicable triangulated framework on the deconstruction of similarly complex social issues using social media data. The exportability of this methodology leads us to propose examining, in similar fashion, data from other Commonwealth nations with colonial pasts and inherited laws criminalizing LGBT communities.
